# Thrombosis in Psoriasis: Cutaneous Cytokine Production as a Potential Driving Force of Haemostatic Dysregulation and Subsequent Cardiovascular Risk

**DOI:** 10.3389/fimmu.2021.688861

**Published:** 2021-07-16

**Authors:** Maria J. E. Visser, Gareth Tarr, Etheresia Pretorius

**Affiliations:** ^1^ Department of Physiological Sciences, Faculty of Science, Stellenbosch University, Stellenbosch, South Africa; ^2^ Division of Rheumatology, Institute of Orthopaedics and Rheumatology, Winelands Mediclinic Orthopaedic Hospital, Stellenbosch, South Africa

**Keywords:** cardiovascular disease, endothelium, hypercoagulability, platelets, psoriasis, systemic inflammation

## Abstract

Psoriasis (PsO) is a common T cell-mediated inflammatory disorder of the skin with an estimated prevalence of 2%. The condition manifests most commonly as erythematous plaques covered with scales. The aetiology of PsO is multifactorial and disease initiation involves interactions between environmental factors, susceptibility genes, and innate and adaptive immune responses. The underlying pathology is mainly driven by interleukin-17. In addition, various inflammatory mediators from specific T helper (T_H_) cell subsets, namely T_H_1, T_H_17, and T_H_22, are overexpressed in cutaneous lesions and may also be detected in the peripheral blood of psoriatic patients. Moreover, these individuals are also at greater risk, compared to the general population, of developing multiple comorbid conditions. Cardiovascular disease (CVD) has been recognised as a prominent comorbidity of PsO. A potential mechanism contributing to this association may be the presence of a hypercoagulable state in these individuals. Inflammation and coagulation are closely related. The presence of chronic, low-grade systemic inflammation may promote thrombosis – one of the major determinants of CVD. A pro-inflammatory milieu may induce the expression of tissue factor, augment platelet activity, and perturb the vascular endothelium. Altogether, these changes will result in a prothrombotic state. In this review, we describe the aetiology of PsO, as well as the pathophysiology of the condition. We also consider its relationship to CVD. Given the systemic inflammatory nature of PsO, we evaluate the potential contribution of prominent inflammatory mediators (implicated in PsO pathogenesis) to establishing a prothrombotic state in psoriatic patients.

## Introduction

Psoriasis (PsO) is a chronic immune-mediated inflammatory disease of the skin, often associated with multiple comorbidities, affecting approximately 2% of the global population (1). Of those affected, 30% may develop an inflammatory arthritis (psoriatic arthritis). The reported prevalence of PsO in childhood may be up to 1.37% (2–4), while the estimated prevalence in adults ranges from 0.51% to 11.43% (3, 5, 6). Research suggests that disease development involves a complex interplay between genetic predisposition, environmental stimuli, and disordered innate and adaptive immune responses. Lesions may assume a variety of clinical forms (7), with plaque PsO being the most common disease variant. Typically, the condition manifests as well-circumscribed, erythematous papules and/or plaques covered with scales. In addition to its physical symptoms, PsO also imposes a significant psychosocial burden that may lead to anxiety, depression, and in severe cases, suicidality (8). The physical and psychological impact of PsO may significantly influence a patients’ quality of life (9), as well as contribute to an increased risk of mortality in severe forms of the disease (10).

PsO is considered to be a T cell-mediated disease and the corresponding cytokine profile of psoriatic lesions indicates important roles for interferon (IFN)-α, interleukin (IL)-22, the IL-23/IL-17 axis, and tumour necrosis factor (TNF) in psoriatic pathology (11–13). In addition to localised cutaneous inflammation, these molecules have also been detected in the systemic circulation of psoriatic patients – increasing the risk of comorbidities (14–16). Considering these observations, a paradigm shift has occurred from viewing PsO as merely ‘skin-deep’ to viewing it as a systemic inflammatory condition that can affect various extracutaneous tissues (17). Cardiovascular disease (CVD) is a notable comorbidity in patients suffering from PsO. An increased risk of major cardiovascular events, as well as an increased CVD mortality in severe forms of the disease, has been reported in these individuals (18–22). The relationship between PsO and CVD is widely acknowledged, however, the mechanisms responsible remain uncertain.

Hypercoagulability is a potential mechanistic link accounting for the association between CVD and PsO. It is becoming increasingly clear that inflammation and coagulation are interrelated processes (23, 24). A hypercoagulable state may develop as a result of an imbalance in haemostatic and inflammatory activity mediated by pro-inflammatory cytokines. These molecules may promote the initiation of coagulation, inhibit endogenous anticoagulant systems, and impair fibrinolytic activity (24, 25). The vascular endothelium also plays a central role in clot formation, as it is located at the nexus of inflammation and coagulation. Under normal physiological conditions, the resting endothelium displays anticoagulant and anti-inflammatory effects (26, 27). This homeostasis is disrupted in inflammatory conditions as a result of pro-inflammatory cytokines perturbing the vessel wall, resulting in a transition to an activated state that favours coagulation (28).

This review will briefly discuss genetic and environmental risk factors associated with the development of PsO, as well as the pathogenesis of the condition. In addition, the literature describing the systemic inflammatory nature of PsO and its subsequent relationship to CVD will also be considered. Finally, the potential contribution of prominent inflammatory mediators in PsO, to promote a prothrombotic state, is also appraised.

## The Aetiology of Psoriasis

### Genetic Risk Factors

The important role of a genetic component in the molecular pathogenesis of PsO has been supported by various family-based studies (29), including twin studies. An increased disease concordance rate has been reported in monozygotic twins, compared to dizygotic twins (30–32). Despite this disease concordance, the incidence never reaches 100% – suggesting a role for environmental factors in addition to genetic susceptibility in PsO development. The current view is that the mode of inheritance for PsO is multifactorial. Classic genome-wide linkage analysis has identified at least nine different chromosomal regions, termed psoriasis susceptibility (*PSORS1*–*PSORS9*) loci, which have shown statistically significant associations with PsO (33). Multiple studies have validated *PSORS1* as the most important genomic region in PsO predisposition and that it may account for up to 35% of disease heritability (34). *PSORS1* is located on human chromosome 6p21.3 within the region of the major histocompatibility complex (MHC). Human leukocyte antigen (HLA)-Cw*0602 has been accepted as the most likely *PSORS1* disease allele (35). The *HLA-C* gene encodes a class 1 MHC protein and participates in the priming of cluster of differentiation (CD) 8^+^ T cell immune responses. More than 80 PsO susceptibility loci have been identified in genome-wide association studies (GWASs), conducted mainly on European and Asian populations (36). In 2007, in the first GWAS for PsO, two genes (*IL12B* and *IL23R*) were identified that were associated with PsO risk (37). Specific pathways implicated in PsO pathogenesis that have been identified through GWASs include interferon signalling, the IL-23 pathway, the nuclear factor kappa-light-chain-enhancer of activated B cells (NF-κB) cascade and the regulation of T cell responses (38–42).

### Environmental Risk Factors

Environmental triggers play a critical role in the onset and development of PsO in genetically predisposed individuals through the interaction with genes and the induction of epigenetic modifications (43). Various environmental exposures have been associated with the initiation and/or exacerbation of psoriatic lesions. Stressful life events are well known to contribute to the initiation and aggravation of PsO. A potential explanation for stress-induced PsO onset or flares may be dysregulation of hypothalamic–pituitary–adrenal axis activity (44, 45). Obesity is another significant risk factor for PsO and body mass index has been demonstrated to correlate with disease severity (46). Potential mediators of this association may include adipokines, such as leptin and resistin, which possess pro-inflammatory actions (47). Furthermore, epidemiological studies have suggested an association between smoking and the development of PsO, based on the increased incidence of PsO reported among current and former smokers compared to non-smokers (48–50). Smoking may induce PsO by augmenting the existing systemic oxidative stress, interacting with immune cells, and altering gene expression (51, 52). Bacterial infections, specifically by *Staphylococcus aureus* and *Streptococcus pyogenes*, are recognised triggers of PsO. β-haemolytic streptococci have been isolated more frequently from throat swabs from PsO patients, compared to controls (53), and enterotoxins from *Staphylococcus aureus* have been linked to more severe PsO (54). Dysbiosis of both the gut and skin microbiome has become a recurrent theme in psoriatic individuals (55). Gut dysbiosis, characterised by a decrease in microbes with anti-inflammatory and immunomodulatory properties (56, 57), seems to be prevalent in these individuals (58, 59). An altered skin microbiome has also been associated with the condition (60, 61).

## Pathogenesis of Psoriasis

Environmental factors (e.g., stress, obesity, smoking, infection, and dysbiosis) or physical trauma (the Koebner phenomenon) may perturb keratinocytes, resulting in the release of self-deoxyribonucleic acid (DNA) or self-ribonucleic acid (RNA) (62, 63). In turn, these self-nucleic acids may form a complex with the endogenous antimicrobial peptide (AMP), cathelicidin/LL37 (63). DNA-LL37 complexes activate plasmacytoid dendritic cells (pDCs), *via* Toll-like receptor (TLR) 9 signalling, which secrete high levels of IFN-α (64). In a similar fashion, RNA-LL37 complexes activate pDCs in a TLR7-dependent manner (62). Consequently, conventional dendritic cells (cDCs), which function as professional antigen-presenting cells (APCs), are activated by IFN-α. RNA-LL37 complexes also possess the capacity to activate cDCs directly, leading to the production of IL-6 and TNF by these cells. Once activated, cDCs migrate to secondary lymphoid organs and secrete IL-12 and IL-23 (65), which induce the differentiation of naïve CD4^+^ T cells into T_H_1 (66) and T_H_17 (67) cells, respectively. This process provides a potential mechanism for the initiation of the inflammatory sequelae in PsO and highlights the central role of DCs to establish a link between the innate and adaptive branches of the immune system.

T_H_1 cells express cytokines such as IFN-γ, IL-2, and TNF (66, 68). IFN-γ induces the expression of several chemokines and cytokines in the skin and also promotes the accumulation and infiltration of inflammatory cells (69, 70). TNF has been identified as a key regulatory molecule in the cytokine network of PsO. In addition to T_H_1 cells, a distinct subset of DCs, namely TNF- and inducible nitric oxide synthase-producing DCs, also release large amounts of TNF (71). It has been suggested that TNF production mediates the proliferation of resident T cells in the development of psoriatic lesions (72). In addition, TNF regulates the expression of cell adhesion molecules, which mediate the extravasation of leukocytes, on endothelial cells and keratinocytes in psoriatic skin (73). TNF also stimulate keratinocytes to induce the expression of pro-inflammatory cytokines such as IL-6, IL-8, and TNF itself, through the activation of NF-κB (74). Furthermore, TNF may synergise with IL-17 to enhance the expression of key inflammatory genes in keratinocytes (75).

T_H_17 cytokines such as IL-17A, IL-17F, and IL-22 (76, 77) are key pathogenic effectors in PsO. Therefore, PsO has been regarded as a T_H_17-mediated disease. However, this paradigm is shifting towards understanding PsO as an IL-17-driven disease (78). Various other cellular sources of IL-17 exist, including CD8^+^ T cells (comprising mucosa-associated invariant T cells and conventional T cells) (79), dermal γδ T cells (80), group 3 innate lymphoid cells (81), mast cells, and neutrophils (82). IL-17A has been shown to govern the expression of signature PsO genes in keratinocytes (83). IL-17 acts on keratinocytes to induce the expression of AMPs, such as β-defensin 2, and neutrophil chemoattractants, namely C-X-C motif chemokine ligand (CXCL)1, CXCL3, CXCL5, CXCL6, and CXCL8 (70). Specifically, IL-17F promotes neutrophil accumulation in the dermis by stimulating the release of IL-8 by keratinocytes (84). T_H_17 cytokines (IL-17A, IL-22, and TNF) also cause keratinocytes to produce C-C motif chemokine ligand (CCL) 20 and its receptor, C-C motif chemokine receptor (CCR) 6, thereby facilitating the recruitment and infiltration of T_H_17 cells (85). IL-17A-induced keratinocyte production of IL-19 has been shown to upregulate the production of antimicrobial proteins, namely S100 calcium-binding protein (S100)A7, S100A8, and S100A9. IL-19 and IL-17A may also interact synergistically to enhance the keratinocyte response. These observations seem to suggest IL-19 as a potentially novel component of the IL-23/IL-17 axis (86), which plays a crucial role in the development of psoriatic inflammation (87). Apart from its role in T_H_17 differentiation and expansion, IL-23 has been demonstrated to induce dermal inflammation and epidermal hyperplasia – mediated through the combined effects of IL-17A (88) and IL-22 (77).

**Figure 1:**
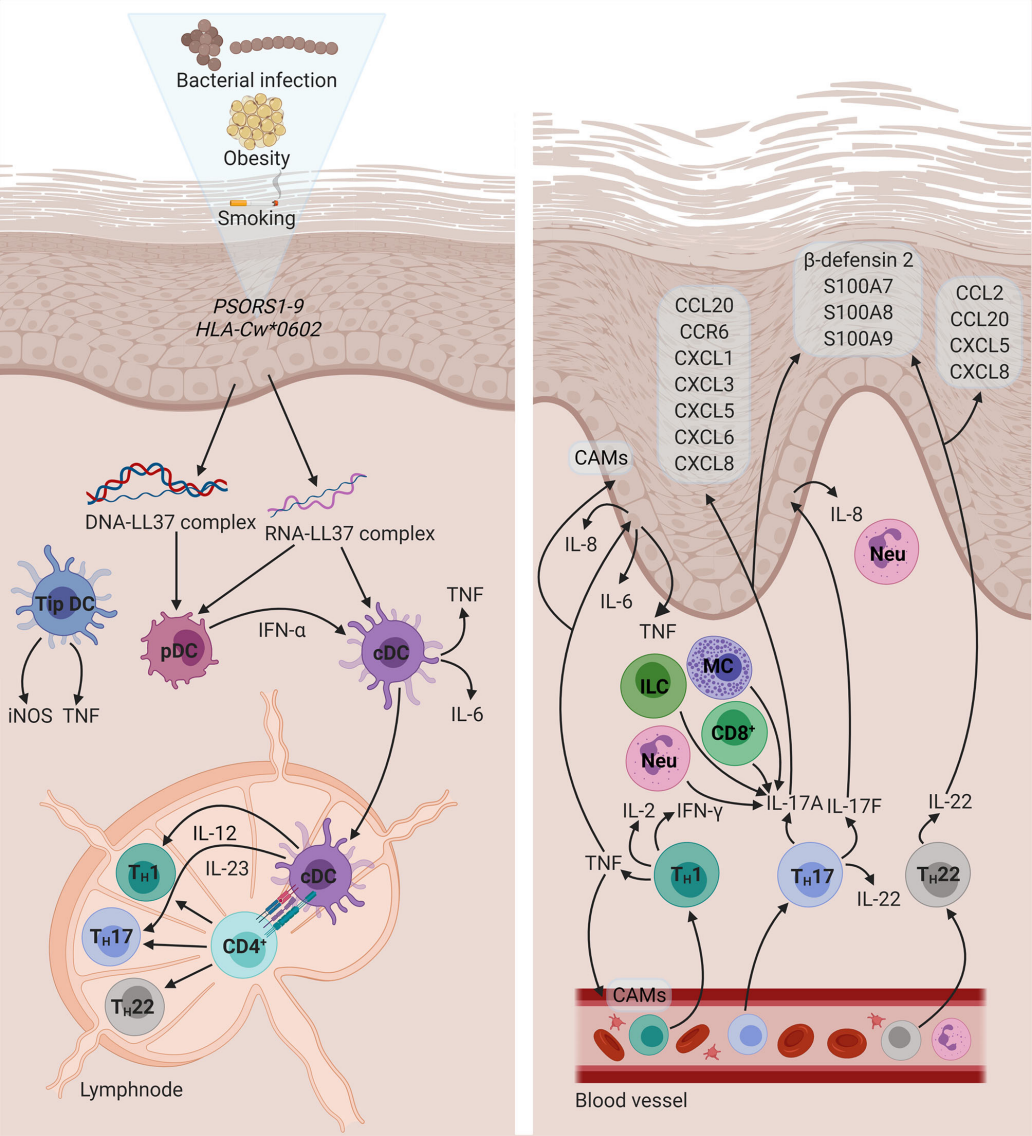
The immunopathogenesis of PsO. A combination of genetic and environmental factors activates pDCs. In turn, cDCs activate naïve CD 4^+^ T cells through the presentation of an unknown antigen. Subsequently, activated CD4^+^ T cells differentiate into T_H_1, T_H_17, and T_H_22 cells, which migrate to the dermis and give rise to a psoriatic plaque. Diagram created with BioRender.com. CAM, cell adhesion molecule; CCL, C-C motif chemokine ligand; CCR, C-C motif chemokine receptor; CD, cluster of differentiation; cDC, conventional dendritic cell; CXCL, C-X-C motif chemokine ligand; DNA, deoxyribonucleic acid; *HLA*, human leukocyte antigen; IFN, interferon; IL, interleukin; ILC, innate lymphoid cell; iNOS, inducible nitric oxide synthase; MC, mast cell; Neu, neutrophil; pDC, plasmacytoid dendritic cell; *PSORS*: psoriasis susceptibility; RNA, ribonucleic acid; S100, S100 calcium-binding protein; T_H_, T helper; Tip DC, tumour necrosis factor- and inducible nitric oxide synthase-producing dendritic cell; TNF, tumour necrosis factor.

IL-22 is co-expressed with IL-17A and IL-17F by activated T_H_17 cells (76, 77) as well as T_H_22 cells (89). IL-22 does not affect immune cells (90) and primarily targets epithelial cells, mediating innate immune responses and contributing to wound healing (91). This cytokine has a dual nature, exhibiting both anti- and pro-inflammatory properties (92). IL-22 induces the overexpression of AMPs, such as β-defensin 2, and the S100 protein family (76, 93, 94). In addition, IL-22 has been reported to downregulate the expression of genes involved in the regulation of keratinocyte differentiation, resulting in acanthosis (94–96). As a pro-inflammatory cytokine, IL-22 stimulates keratinocyte production of chemokines (CCL2, CCL20, CXCL5, and CXCL8) (93, 94). These chemotactic agents will promote the infiltration of monocytes/macrophages (97), neutrophils (98), and T cells (99) at sites of cutaneous inflammation. A graphical representation of key cytokine circuits in the immunopathogenesis of PsO is provided in **Figure 1**.

Other cytokines that have been implicated in psoriatic inflammation include IL-9, IL-19, IL-20, IL-24, IL-33, and IL-36. IL-9 has been suggested to contribute to PsO pathogenesis *via* its pro-angiogenic activity and induction of IL-17 production (100). In addition, members of the IL-20 subfamily of cytokines (IL-19, IL-20, and IL-24) have been shown to affect keratinocyte proliferation and differentiation and to induce the expression of various PsO-related molecules (101). Indeed, suppression of these cytokines resulted in alleviation of epidermal hyperplasia in psoriatic patients (102). Furthermore, IL-33 was recently demonstrated to act on keratinocytes in an autocrine manner, thereby perpetuating the psoriatic inflammatory response (103). Finally, IL-36 has been implicated in keratinocyte-specific pathways that mediate dermal inflammation in PsO (104, 105).

## Treatment of Psoriasis

When determining the appropriate treatment regimen for a PsO patient, various factors should be taken into consideration, such as disease severity the presence of psoriatic arthritis, comorbidities, and the impact on the patient’s quality of life. The severity of PsO may be determined by the percentage of the total body surface area (BSA) that is involved, with <3% BSA considered mild, 3–10% BSA considered moderate, and >10% BSA considered severe. Mild to moderate PsO is treated with topical agents including corticosteroids, calcineurin inhibitors, vitamin D_3_ analogues, keratolytic agents, anthralin, retinoids, and coal tar preparations (106). For moderate to severe PsO, phototherapy and systemic treatments are prescribed (107). Oral systemic therapies, such as methotrexate, apremilast, cyclosporine, and acitretin, possess anti-inflammatory and immunomodulatory properties (108). The advent of biologic systemic therapies has drastically changed the treatment of PsO. Currently, there are four classes of biologicals available, namely TNF inhibitors, IL-12/23 inhibitors, IL-17 inhibitors, and IL-23 inhibitors (109). These agents exert their effects by targeting prominent cytokines involved in the pathogenesis of PsO. When compared to traditional systemic agents these therapies have better safety profiles and may also be more efficacious (110).

## The Systemic Inflammatory Nature of Psoriasis

PsO was initially primarily regarded as a hyperkeratotic disorder solely confined to affected skin areas. However, the systemic inflammatory nature of the condition has become increasingly apparent in recent years (111, 112). Various pro-inflammatory products are overexpressed in psoriatic skin lesions. These mediators also seem to be released into the systemic circulation of psoriatic patients and may reflect disease severity (113). Peripheral inflammation is evident by the abnormal expression of a host of inflammatory molecules in the blood of these individuals (113–116). Moreover, inflammation may also be detected at extracutaneous sites. In a pilot study by Mehta et al. (117), ^18^F-fluorodeoxyglucose emission tomography-computed tomography was utilised to localise and quantify inflammatory activity in individuals with moderate to severe PsO. The authors detected systemic inflammation in the skin, joints, liver, and vasculature, with significantly greater aortic and hepatic inflammation in psoriatic patients compared to age- and gender-matched controls (117). Furthermore, individuals with PsO have an increased risk of developing multiple comorbid diseases (presented in **Table 1**), which serves as further evidence for the presence of systemic inflammation in the condition. It has been proposed that the chronic course of PsO, as well as common inflammatory molecules and/or pathways, may act as the driving forces of the development and/or worsening of these extracutaneous manifestations (15, 111).

**Table 1:** Comorbidities associated with PsO.

Comorbidity	Selected references
Chronic kidney disease	(118–120)
CVD	(18–20)
Depression	(8, 121, 122)
Diabetes	(123–125)
Inflammatory bowel disease	(126–128)
Malignancy	(129–131)
Metabolic syndrome	(132–134)
Non-alcoholic fatty liver disease	(135–137)

CVD, cardiovascular disease.

Besides systemic comorbid conditions, individuals with PsO may also develop metabolic abnormalities. An increased prevalence of metabolic syndrome (MetS) – a constellation of dyslipidaemia, hypertension, insulin resistance, and visceral obesity – has been reported in psoriatic patients compared to healthy controls (132, 133, 138–140). However, the directionality of these associations remains unknown. It is possible that PsO may initiate inflammatory pathways that drive the development of metabolic disturbances. Inflamed adipose tissue produces adipocytokines (141) that may worsen existing PsO (142). We note that metabolic dysfunction may also contribute to an increased cardiovascular burden in psoriatic individuals. This is however, beyond the scope of this paper. There are several excellent review articles in which the association between MetS and PsO are discussed (143–145).

## The Association Between Psoriasis and Cardiovascular Disease

In recent years, CVD has emerged as a particularly prominent comorbidity of PsO. Several epidemiological studies have reported an increased risk of CVD in psoriatic individuals, compared to the general population. Furthermore, recent guidelines for the primary prevention of CVD, by the American College of Cardiology and the American Heart Association, indicated PsO as a risk-enhancing factor for the development of atherosclerotic CVD (146).

In a landmark study by Gelfand and colleagues (20), patients with PsO were reported to have a higher incidence of myocardial infarction (MI). Patients with mild and severe PsO had an incidence of 4.04 (95% confidence interval [CI]: 3.88–4.21) and 5.13 (95% CI: 4.22–6.17) per 1000 person-years, compared to an incidence of 3.58 in healthy control subjects (95% CI: 3.52–3.65) (20). In addition, traditional CVD risk factors such as hyperlipidaemia, hypertension, and smoking were more prevalent among psoriatic individuals. PsO remained an independent risk factor for CVD, after adjusting for established CVD risk factors. Similar associations were noted in studies assessing the risk of stroke (19) and venous thromboembolism (VTE) (18) in psoriatic individuals. Gelfand et al. (19) reported an increased risk of stroke in mild (hazard ratio [HR] 1.06; 95% CI: 1.0–1.1) and severe (HR 1.43; 95% CI: 1.1–1.9) PsO, after adjusting for major risk factors (19). Furthermore, Ahlehoff and colleagues (18) reported higher incidence rates of VTE in psoriatic individuals (1.92 and 3.20 per 1000 person-years for mild and severe PsO) compared to healthy controls (1.29 per 1000 person-years) (18). In a meta-analysis by Gaeta and colleagues (147), it was shown that PsO confers an excess risk of 24% for the development of CVD (147). It has also been reported that severe disease activity contributes substantially to increased mortality due to CVD (148–150). Mehta et al. (21) identified severe PsO as an independent risk factor for CVD deaths (HR 1.57; 95% CI: 1.26–1.96), after adjusting for traditional CVD risk factors (21). Lastly, in a study by Abuabara and colleagues (149), it was determined that CVD was responsible for the highest absolute (61.9 deaths per 1000 person-years) and excess (3.1 deaths per 1000 person-years) risk in PsO patients (149).

The link between PsO and CVD may potentially be explained by the chronic course of the disease and the associated systemic inflammation. A considerable body of literature supports the notion that chronic low-grade systemic inflammation is a central theme in the pathogenesis and propagation of CVD (151–154). Moreover, elevated levels of C-reactive protein (CRP) – a sensitive marker of systemic inflammation – has been suggested as a predictor of future CVD events (155). Two landmark trials, namely the Justification for the Use of Statins in Prevention: an Intervention Trial Evaluating Rosuvastatin (JUPITER) study (156), and the Canakinumab Anti-inflammatory Thrombosis Outcome Study (CANTOS) (157), also provided evidence for a prominent role of systemic inflammation in the etiopathogenesis of CVD. In the following section, we will elaborate on the interplay between inflammation and coagulation as well as how an imbalance in these activities may promote the development of CVD – specifically *via* thrombosis.

## Psoriatic Inflammation and Coagulopathy

Inflammation and coagulation are interdependent processes, demonstrated by the dynamic crosstalk between these systems. Under normal physiological conditions, these systems function as protective mechanisms and are tightly regulated. However, dysregulation may result in chronic, systemic inflammation and/or thrombotic complications. In response to invading pathogens or tissue damage, inflammation ensues to eliminate the original insult and to promote wound healing and tissue repair. However, if the inflammatory process is not duly resolved; acute inflammation may transition to chronic, systemic inflammation. In turn, the sustained activation of the coagulation cascade, driven primarily by pro-inflammatory cytokines, may follow.

A particularly prominent molecule in the context of inflammation-induced coagulation is tissue factor (TF) (25). The tenase complex, comprising TF and factor VIIa, activates the extrinsic coagulation pathway, culminating in the generation of thrombin. In the final steps of coagulation, thrombin catalyses the conversion of soluble fibrinogen to insoluble fibrin. Pro-inflammatory cytokines may upregulate the expression of TF on endothelial cells and monocytes (158). In addition, inflammation favours the suppression of natural anticoagulant mechanisms, namely the antithrombin pathway, the protein C pathway, and tissue factor pathway inhibitor (TFPI) (24, 25). Finally, fibrinolytic activity may also decrease due to a continuous increase in plasminogen activator inhibitor (PAI)-1 levels stimulated by pro-inflammatory molecules (25). Thus, chronic systemic inflammation may alter the haemostatic balance to favour a prothrombotic state.

In turn, coagulation may also modulate and perpetuate the inflammatory response. Coagulation proteases may bind to protease-activated receptors on the activated endothelium, inducing the synthesis and expression of cell adhesion molecules (159, 160). These molecules play a pivotal role in the extravasation of leukocytes to sites of inflammation. Furthermore, activated coagulation factors may also elicit an inflammatory response by interacting with immune cells to induce the production of cytokines (161, 162). Thrombocytes or platelets are also increasingly recognised for their ability to mediate and regulate inflammation. Activated platelets release their granular content, which comprises a plethora of procoagulant and pro-inflammatory molecules. Platelets are also implicated in the recruitment of leukocytes and the regulation of vascular permeability (163).

From these observations, it is evident that inflammation and coagulation should not be viewed as separate entities and that an imbalance in these activities may culminate in the development of prothrombotic conditions. Thrombosis is the most common pathology underlying the three major cardiovascular conditions, namely ischaemic heart disease, ischaemic stroke, and VTE (164–166). Arterial thrombosis, which ensues after the rupture of an atherosclerotic plaque or damage to the vessel wall, may give rise to MI or stroke. Platelets play a central role in the formation of an arterial thrombus (167). In contrast, the pathomechanism of venous thrombosis, associated with deep vein thrombosis (DVT) and pulmonary embolism, is less clear. Virchow’s triad describes three factors, namely hypercoagulability, endothelial dysfunction, and altered blood flow, which may predispose an individual to the development of venous thrombosis (168).

**Figure 2:**
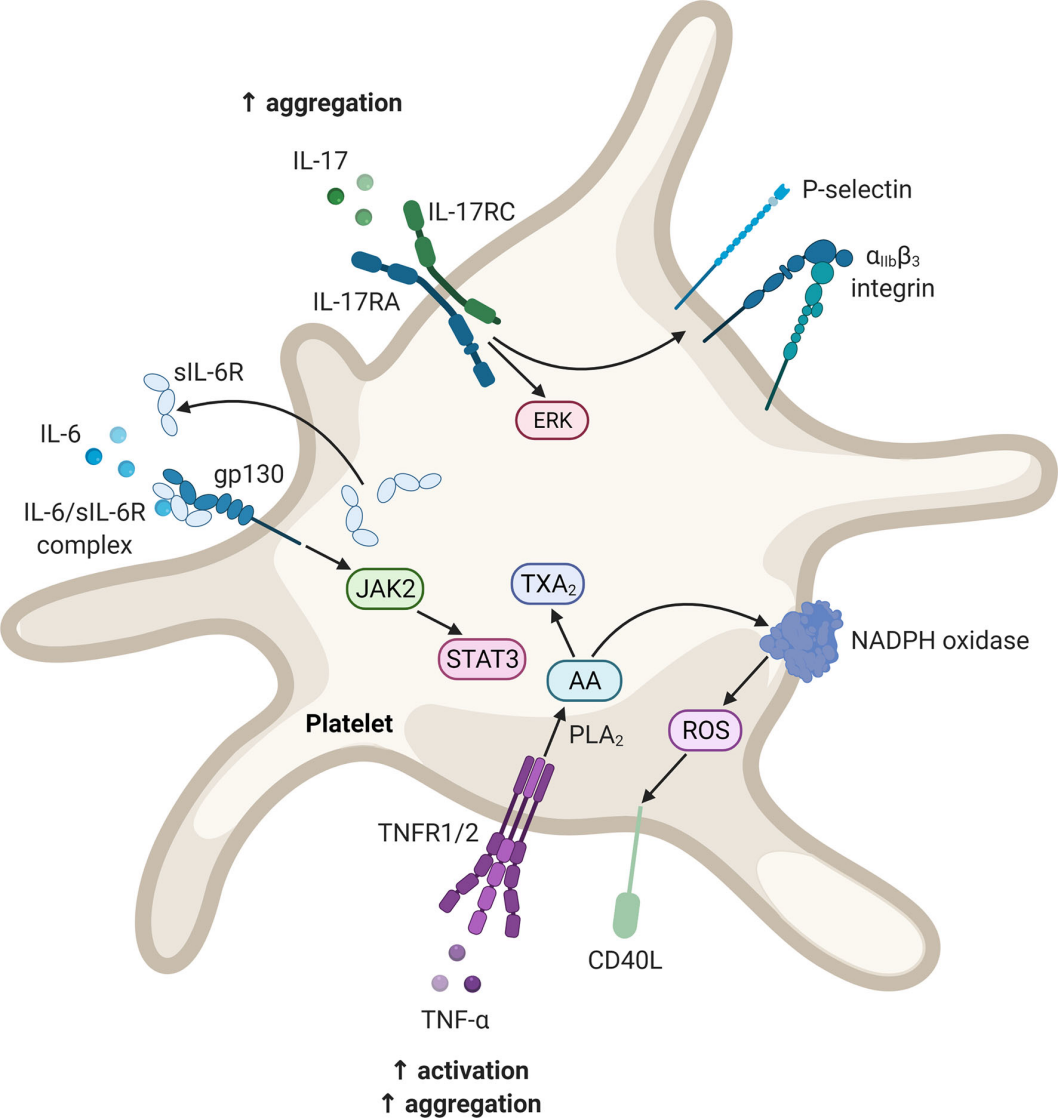
Potential signalling pathways in platelets upon stimulation with IL-6, IL-17, and TNF. These mediators may contribute to platelet activation as well as enhancing the platelet response to agonist-induced platelet activation. Diagram created with BioRender.com. AA, arachidonic acid; CD40L, CD40 ligand; ERK, extracellular signal-regulated kinase; gp, glycoprotein; IL, interleukin; IL-17RA, interleukin-17 receptor A; IL-17RC, interleukin-17 receptor C; JAK, Janus kinase; NADPH, nicotinamide adenine dinucleotide phosphate; PLA_2_, phospholipase A_2_; ROS, reactive oxygen species; sIL-6R, soluble interleukin-6 receptor; STAT, signal transducer and activator of transcription; TXA_2_, thromboxane A_2_; TNF, tumour necrosis factor; TNFR, tumour necrosis factor receptor.

**Figure 3:**
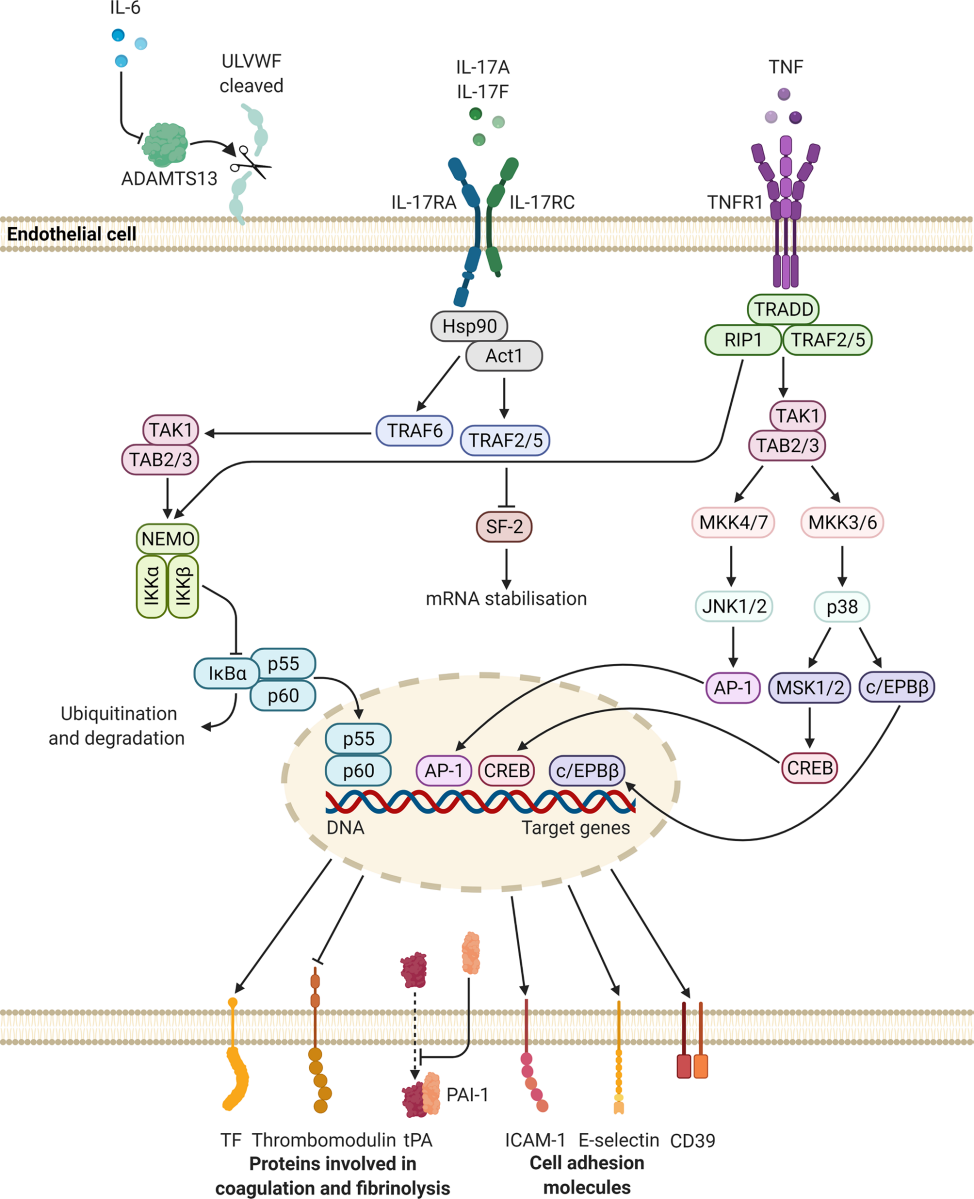
Potential intracellular signalling pathways initiated in endothelial cells, upon stimulation with IL-6, IL-17, and TNF. These mediators may upregulate the synthesis and release of molecules that promote platelet adhesion and coagulation, while suppressing the expression of proteins involved in fibrinolysis. Altogether these changes may lead to a prothrombotic state. Diagram created with BioRender.com. ADAMTS13, a disintegrin-like and metalloprotease with thrombospondin type 1 motif, 13; AP, activator protein; c/EPB, CCAAT/enhancer-binding protein; CD, cluster of differentiation; CREB, cAMP response element binding protein; DNA, deoxyribonucleic acid; Hsp, heat shock protein; ICAM-1, intercellular adhesion molecule-1; IKBα, NF-kappa-B inhibitor alpha; IKK, inhibitor of nuclear factor kappa-B kinase; IL, interleukin; IL-17RA, interleukin-17 receptor A; IL-17RC, interleukin-17 receptor C; JNK, c-Jun N-terminal kinase; MKK, mitogen-activated protein kinase kinase; MSK, mitogen- and stress-activated protein kinase; NEMO, NF-kappa-B essential modulator; PAI-1, plasminogen activator inhibitor-1; SF, splicing factor; TF, tissue factor; TNF, tumour necrosis factor; TNFR, tumour necrosis factor receptor; tPA, tissue-type plasminogen activator; TRADD, tumour necrosis factor receptor 1-associated death domain protein; TRAF, tumour necrosis factor receptor-associated factor; RIP, receptor-interacting protein kinase; ULVWF, ultra-large von Willebrand factor.

As discussed in the previous sections, PsO is characterised by chronic, systemic inflammation and accompanied by an increased risk for CVD. A potential mechanism may be the development of a prothrombotic state, *via* the action of multiple pro-inflammatory cytokines, due to psoriatic pathology. The most prominent cytokines that could play a role in the development of a prothrombotic state - IL-6, IL-17, and TNF - will be discussed below. We focus specifically on the effects of these cytokines on platelets and the endothelium. In **Figures 2** and **3** schematic representations are provided of the signalling pathways that could be initiated in platelets and endothelial cells, respectively, upon stimulation with these cytokines.

### Interleukin-6

IL-6 is a pleiotropic cytokine involved in several physiological processes including the acute-phase response and antibody production (169). The effects of IL-6 are mediated *via* two receptors, namely the IL-6 receptor (IL-6R) and the signal-transducing subunit, glycoprotein (gp) 130 (169). In PsO, IL-6, combined with IL-23, contributes to the differentiation of T_H_17 cells (12). The overexpression of this cytokine has been detected in psoriatic skin lesions (170) as well as in the circulation of these individuals (113, 171, 172). In addition, IL-6 signalling has been suggested to diminish regulatory T cell activity in PsO, thereby allowing the expansion of effector T cells (170). In the context of CVD, IL-6 has emerged as a pivotal mediator of thrombotic disease. Raised IL-6 levels have been found to be related to recurrent venous thrombosis (173), with detectable levels of IL-6 associated with a two-fold increase in the risk of venous thrombosis (174). The potential involvement of IL-6 in the development of adverse cardiovascular events is further supported by findings of a sub-study of the CANTOS trial. In this study, baseline IL-6 levels were associated with an increased risk of cardiovascular events. More importantly, it was demonstrated for the first time that lowering IL-6 levels – *via* the inhibition of IL-1β by canakinumab – resulted in a 15% reduction of atherothrombotic events (MI and stroke) (175).

During inflammation, IL-6 acts on hepatocytes to induce the synthesis of acute-phase proteins such as CRP, fibrinogen, and serum amyloid A (SAA) (176). Both CRP and SAA have demonstrated the ability to promote coagulation *via* the induction of TF synthesis in endothelial cells and suppression of the TFPI pathway (177, 178). With regard to fibrinogen, it has been suggested that a causal relationship exists between hyperfibrinogenaemia and thrombosis (179).

Although platelets do not express IL-6R, they have been shown to express gp130 (180, 181). Upon thrombin-induced platelet activation, the soluble form of IL-6R is secreted which can form a complex with IL-6 (180, 181). This complex may activate gp130, in a process termed trans-signalling, resulting in the activation of signal transducer and activator of transcription (STAT) 3 (181). However, IL-6 trans-signalling does not seem to affect platelet activation and/or aggregation. Nevertheless, IL-6 may promote platelet adhesion and aggregation indirectly. IL-6 inhibits the activity of a disintegrin-like and metalloprotease with thrombospondin type 1 motif, 13 (ADAMTS13) (182). ADAMTS13 is responsible for the cleavage of ultra-large von Willebrand factor multimers released by the endothelium. In the ultra-large form, these multimers are hyperreactive and may interact with platelets to induce adhesion and aggregation.

### Interleukin-17

IL-17 plays a pivotal role in the innate immune response, particularly in host defence against microbial invasion (183). The IL-17 family consists of six members, namely IL-17A to IL-17F, and signalling is mediated through the IL-17 receptor (IL-17R) family that comprises five members, IL-17RA to IL-17RE (184). This pro-inflammatory cytokine has been implicated in the pathogenesis of diverse autoimmune and inflammatory diseases, including PsO (185). Indeed, IL-17 is regarded as the principal driver of psoriatic inflammation. The overexpression of this cytokine has been detected in lesional psoriatic skin, when compared to nonlesional skin (186, 187). In addition, serum levels of IL-17A are elevated in psoriatic patients (188) and may also reflect disease severity (186). With respect to cardiovascular pathology, conflicting results have been reported on the role of IL-17. Some studies have found a protective effect of IL-17 (189), while others have reported a pro-atherogenic role for IL-17 (190). Nevertheless, prothrombotic effects of IL-17 have been demonstrated. In a murine model of DVT, IL-17A was found to promote thrombus formation by enhancing platelet aggregation and neutrophil infiltration of thrombi (191). Moreover, it has been shown that targeted blocking of IL-12/23 and IL-17 resulted in improved skin phenotype and lengthened clotting times to occlusive thrombus formation in a murine model of psoriatic disease (192).

Platelets have been demonstrated to express a functional IL-17 receptor, namely IL-17RA (193). Exposing activated platelets (induced by adenosine diphosphate) to IL-17 augments platelet aggregation (193–195). This action is suggested to be mediated by the opening of the mitochondrial permeability transition pore (196) and the phosphorylation of extracellular signal-regulated kinase-2 (194, 196). Furthermore, stimulation with IL-17 causes an increased expression and accelerated externalisation of P-selectin and exposure of the α_IIb_β_3_ integrin (193, 194, 196). Both of the aforementioned molecules facilitate platelet aggregation and are used as markers of platelet activation.

IL-17 may also alter the endothelium towards an activated state. IL-17RA is constitutively expressed in endothelial cells (197). It has been reported that IL-17 and TNF act synergistically to activate the endothelium, resulting in the synthesis of inflammatory mediators, and the expression of cell adhesion molecules (E-selectin and intercellular adhesion molecule-1) and TF by these cells (195). Anticoagulant activity by the endothelium also becomes diminished, as thrombomodulin expression is downregulated (195). It should be noted that IL-17 alone is not a strong inducer of inflammatory activity, however, IL-17 in concert with TNF potently induce pro-inflammatory gene expression. This effect is thought to be mediated by the ability of IL-17 to stabilise messenger RNA (198, 199). Finally, the vascular expression of CD39, an inhibitor of platelet aggregation, is also downregulated by IL-17A (195, 200).

### Tumour Necrosis Factor

TNF is a potent inflammatory cytokine, orchestrating various processes such as inflammation, cell differentiation, and apoptosis (201). The effects of TNF are mediated by two receptors, namely TNF receptor (TNFR) 1 and TNFR2 (202). In PsO, this cytokine amplifies the inflammatory response *via* the generation of reactive oxygen species and inducing the expression of cytokines and cell adhesion molecules (73, 203). Increased TNF activity has been detected in involved skin from psoriatic individuals (204) and TNF levels were also elevated in the serum of these individuals (113, 205). In relation to cardiovascular diseases, increased concentrations of TNF have been associated with recurrent coronary events (206), and venous thrombosis (174). In addition, TNF levels have been suggested to be an independent predictor of cardiovascular events such as MI, stroke, and CVD mortality (207). Regarding the prothrombotic properties of TNF, discrepant results have been reported. In a murine model of atherothrombosis, potent antithrombotic effects of TNF were reported (208). In contrast, another study found that treatment with this cytokine resulted in accelerated thrombus formation (209).

It has been suggested that TNF may promote the (hyper)activation of platelets by interacting with TNFR1 and TNFR2, which is expressed on the platelet membrane (210). TNF may also facilitate platelet activation by stimulating the arachidonic acid pathway (210). Furthermore, CD40 ligand (CD40L) expression by activated platelets may be induced by TNF *via* an arachidonic acid-dependent oxidative stress mechanism (211, 212). In turn, CD40L enhances platelet activation and aggregation, as well as thrombus formation (213). TNF also induces the release of large von Willebrand factor multimers from endothelial cells, which propagates platelet thrombus formation (182). Nevertheless, the ability of TNF to stimulate platelet activation directly remains contested, with some studies reporting no effect (209).

TNF may alter the properties of the endothelium by inducing the synthesis of procoagulant molecules and suppressing natural anticoagulant mechanisms. Engagement of TNF with TNFR1 results in the expression of TF on the surface of endothelial cells and the subsequent deposition of fibrin (209, 214–216). In addition, the production of platelet-activating factor (PAF), by the endothelium, is also induced by TNF (217, 218). PAF induces platelet aggregation (219) and functions as a potent inflammatory mediator (220). The activity of the anticoagulant molecule, activated protein C, is dependent on the presence of functional endothelial thrombomodulin, which has been shown to be downregulated by TNF (216). Mechanistically, this inhibitory effect is mediated by downregulating the transcription of the thrombomodulin gene (209, 221). Finally, TNF inhibits fibrinolysis by induction of the PAI-1 gene (209) through the activity of NF-κB (222) and by decreasing the release of tPA (223).

## Concluding Remarks

**Figure 4:**
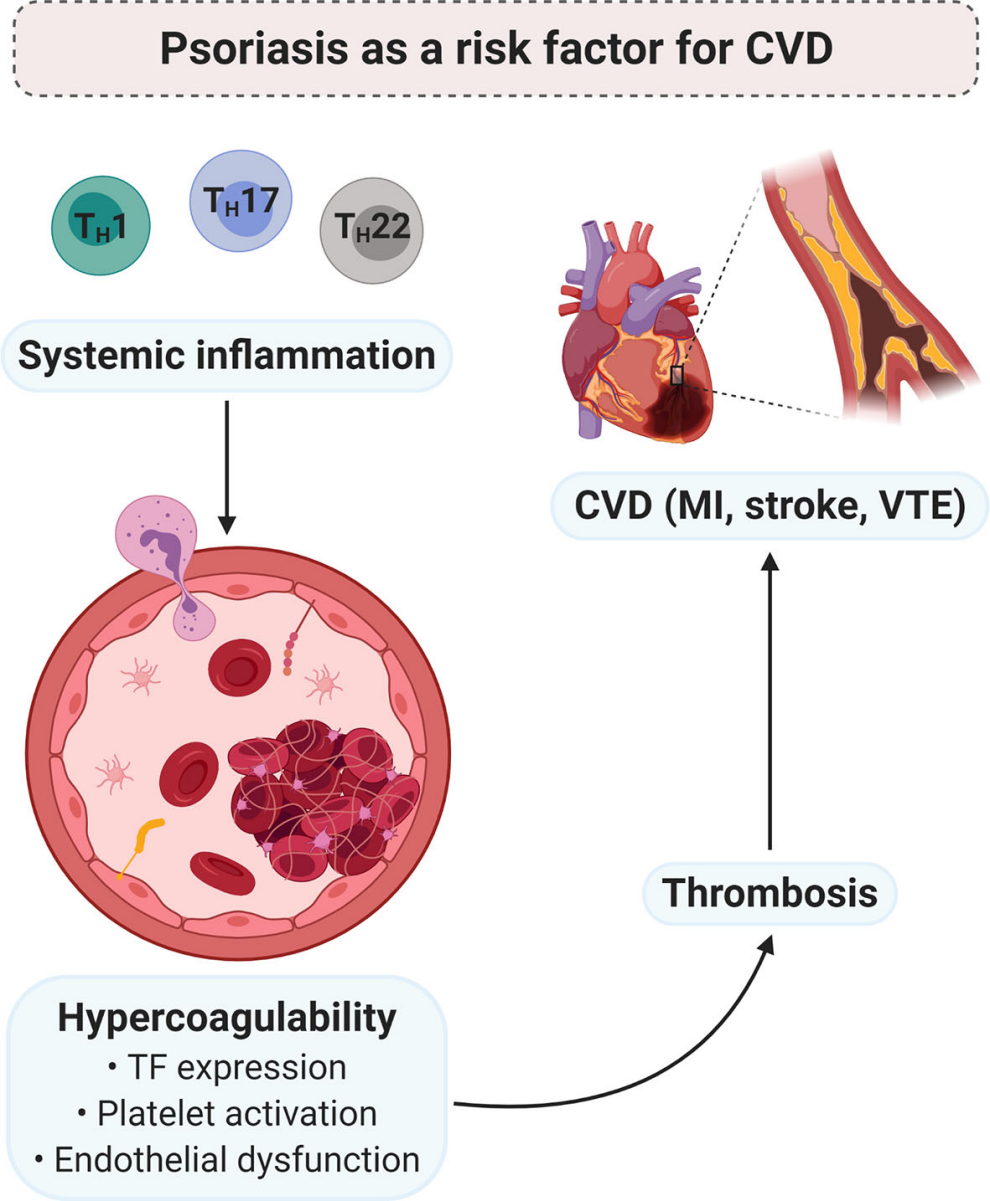
PsO as a risk factor for CVD. Chronic low-grade systemic inflammation, due to psoriatic pathology, may contribute to the development of a hypercoagulable state. Pro-inflammatory mediators induce the expression of TF, enhance platelet activation and/or the platelet response, and endothelial dysfunction. In turn, hypercoagulability predisposes thrombosis, which is the major underlying pathology of CVD. Diagram created with BioRender.com. CVD, cardiovascular disease; MI, myocardial infarction; TF, tissue factor; T_H_, T helper; VTE, venous thromboembolism.

PsO is an immune-mediated inflammatory disorder of the skin, characterised by the overexpression of T_H_1-, T_H_17-, and T_H_22-derived inflammatory cytokines. These mediators are upregulated in the lesional skin of psoriatic individuals and may also be released into the circulatory system of these patients. The systemic inflammatory nature of the condition is reflected by a multitude of dysregulated inflammatory molecules, which may give rise to various comorbidities. PsO has been identified as an independent risk factor for the development of CVD (**Figure 4**). The chronic subclinical systemic inflammation associated with the condition may predispose psoriatic individuals to thrombosis – a major determinant of CVD. Inflammatory cytokines may contribute to the development of a prothrombotic state, *via* the induction of TF, platelet activation and/or enhancing the platelet response, and endothelial dysfunction.

The exact mechanisms underlying the association between PsO and CVD remains elusive. However, it is imperative that both physicians and patients must be aware of the potential cardiovascular risk that PsO may pose. Therefore, effective management of the condition should not only aim to ameliorate cutaneous inflammation but also systemic inflammation, in order to prevent secondary comorbidities such as CVD. Finally, as pointed out in a recent review by Aksentijevich and colleagues (224), PsO presents a unique opportunity as a human model for the study of chronic systemic inflammation in the development of CVD (224). Treatment of psoriatic patients with targeted biological therapies may shed light on the contribution of specific cytokines to cardiovascular morbidity, and provide novel treatment targets.

## Author Contributions

MV: writing of paper, and preparation of figures. GT: editing of paper. EP: study leader, corresponding author, editing of paper, funding. All authors contributed to the article and approved the submitted version.

## Funding

This work is based on the research supported in part by the National Research Foundation (NRF) of South Africa (Grant Numbers: 117473 and 132825). The funders had no role in study design, data collection and analysis, decision to publish, or preparation of the manuscript.

## Conflict of Interest

The authors declare that the research was conducted in the absence of any commercial or financial relationships that could be construed as a potential conflict of interest.
